# What Remains up to 7 Months after Severe and Moderate Pneumonia in Non-Vaccinated Patients with Long COVID? Results of a CT Study

**DOI:** 10.3390/jcm12165388

**Published:** 2023-08-19

**Authors:** Ewa Kurys-Denis, Anna Grzywa-Celińska, Katarzyna Podgórska, Miłosz Piotr Kawa

**Affiliations:** 1 2nd Department of Radiology, Medical University of Lublin, 20-059 Lublin, Poland; 2 Chair and Department of Pneumonology, Oncology and Allergology, Medical University of Lublin, 20-059 Lublin, Poland; 3 Department of Diagnostic Imaging, Center of Oncology of the Lublin Region St. Jana z Dukli, 20-090 Lublin, Poland; 4 Department of General Pathology, Pomeranian Medical University, 70-204 Szczecin, Poland; 5 Department of General and Dental Diagnostic Imaging and Interventional Radiology, Pomeranian Medical University, 70-111 Szczecin, Poland

**Keywords:** SARS-CoV-2, COVID-19, lung abnormalities, chest CT, 7 months, mean 5 months, 4 months, convalescent patients

## Abstract

Background: There is a growing evidence of long-lasting lung changes after COVID-19. Our aim was to assess the degree of lung injury and evaluate the recovery process of 4–7-month-non-vaccinated convalescent patients discharged from hospital after moderate and severe COVID-19 pneumonia, who presented with symptoms of long-COVID. Methods: On control lung CT after mean 5-month recovery period, we classified and determined the prevalence of residual radiological abnormalities in 39 symptomatic patients. To assess the advancement of the persisting changes we used the total severity score (TSS) and the chest CT score and then correlated the results with clinical data. Results and conclusions: On follow-up CT images, 94.9% of patients showed persistent radiological abnormalities. The most frequent changes were ground-glass opacities (74.4%), reticular pattern (64.1%), fibrotic changes (53.8%), nodules (33.3%), bronchiectasis (15.4%), vascular enlargement (10.3%), and cavitation (5.1%). The median TSS score was 4.1 points (interquartile range 3), whereas the median of the chest CT score 5.4 points (interquartile range of 4.5). No significant differences were observed between sex subgroups and between the severe and moderate course groups. There were no association between both CT scores and the severity of the initial disease, indicating that, mean 5 months after the disease, pulmonary abnormalities reduced to a similar stage in both subgroups of severity.

## 1. Introduction

Severe acute respiratory syndrome coronavirus 2 (SARS-CoV-2) identified in January 2020 as the etiological factor of the Coronavirus Disease (COVID-19) pandemic pneumonia belongs to the family of Coronaviridae. Along with the previously described pathogens: severe acute respiratory syndrome coronavirus 1 (SARS-CoV-1) and Middle East respiratory syndrome coronavirus (MERS-CoV), all three cause severe respiratory infections. Although the clinical course of COVID-19 is variable, from asymptomatic, through severe pneumonia to multi organ failure, the predominant feature of infection is lung damage [1,2,3,4]. SARS-CoV-2 primarily targets both the upper and lower segments of the respiratory system. Within the lungs, the virus predominantly localizes in pneumocytes and macrophages, triggering inflammation, fluid accumulation, and injury to pulmonary tissues. This clinical manifestation results in symptoms such as dyspnea, pneumonia, and, in the final stage, acute respiratory distress syndrome (ARDS). Currently, as the acute phase of the SARS-CoV-2 pandemic has come to an end, we are still facing the challenge of its long-term effects. Many patients may experience persistent symptoms that continue to impact their quality of life and may lead to the development of further complications, such as cardiovascular dysfunctions in the course of chronic lung diseases. Early identification of these effects allows for earlier treatment and the limitation of potential complications. Knowledge about the long-term consequences of pneumonia in COVID-19 can contribute to the improvement of treatment strategies and medical care.

Computed tomography (CT) imaging has played a significant role in diagnosing pneumonia in the course of COVID-19 since the beginning of the pandemic. It allowed for early detection of characteristic changes associated with this disease, which was particularly valuable during the initial stages of the pandemic when the number of available tests was insufficient and the waiting time for results was long. In many cases, this enabled faster isolation and implementation of appropriate measures to prevent further virus spread. One of the crucial applications of CT imaging is to assess the extent and severity of inflammatory changes in the lungs, which is vital to understand the patient’s condition and make decisions regarding treatment. Additionally, CT’s role includes ruling out other diseases with similar symptoms.

With the growing number of survivors, one of the most pressing concerns is currently researching possible post-COVID sequelae. Long COVID, also known as post-acute COVID syndrome, is a condition affecting individuals with a history of confirmed or probable COVID-19 infection and comprises a wide variety of symptoms, physical and psychological, sometimes lowering quality of life. Symptoms are often nonspecific, such as fatigue and dyspnea, and difficult to assess objectively. In cases of comorbidity with preexisting cardiopulmonary diseases, there is also an overlap with symptoms [5]. There has been growing evidence of residual lung changes in convalescent COVID-19 patients in the long term [6,7,8,9,10,11]. However, few studies classify and correlate persistent abnormalities with a distinct course of the SARS-CoV-2 infection with CT scores.

### Aim of the Study

In our work, we aimed to assess the degree of lung injury and evaluate the recovery process of 4–7-month non-vaccinated convalescent patients discharged from the hospital after moderate and severe COVID-19 pneumonia by classifying and determining the prevalence of residual radiological lung abnormalities. Additionally, we attempted to evaluate the advancement of persisting radiological changes using Kunwei Li’s total severity score (TSS) and Kunhua Li’s chest CT score. Moreover, our objective was to assess the CT abnormalities concerning the disease’s onset severity during hospitalization, age, gender, and duration of recovery.

## 2. Materials and Methods

Out of 165 initially enrolled patients, hospitalized for moderate or severe COVID-19 pneumonia between 1 September 2020 and 15 April 2021, only 39 patients turned up for a control CT examination 4–7 months (mean 5 months) after being discharged from the hospital (92 declined to participate as they were feeling well, 14 patients died from the disease, and 20 were unable to be contacted 7 months after the disease). All patients received treatment based on the prevailing clinical guidelines. Before leaving the hospital, they all met standard discharge criteria, including >3 days of normal body temperature, improvement of laboratory and clinical symptoms and 2 negative consecutive SARS-CoV-2 tests. Chest CTs were performed at a follow-up control examination performed between 15 March 2021 and 31 October 2021 on patients who complained of at least one common symptom classified for long COVID-19 [12]. Patients with pre-existing chronic respiratory or psychiatric conditions were not included in the study. Our retrospective cohort study group was classified into two subgroups of moderate (*n* = 16) and severe course of the disease (*n* = 23), according to the recommendations of Polish Association of Epidemiologists and Infectiologists [13]. Patients were qualified to the moderate initial course of the disease if they fulfilled the clinical criteria for stage 2 or 3 of COVID-19 (full symptoms of viral replication or cytokine storm, SpO_2_ < 95% or SpO_2_ < 90). The severe group of patients included those who developed acute respiratory distress syndrome (ARDS) despite earlier pharmacotherapy and needed mechanical ventilation during hospitalization [13].

Our protocol for high resolution computed tomography (HRCT) imaging included scanning lungs from apices to bases in the supine position during deep inspiration and breath holding. No intravenous contrast was used. We used a General Electric 64-row scanner (GE Healthcare, Waukesha, WI, USA). The following parameters were used during the CT scanning: 120 kVp of tube voltage, tube current of 650 mA with automatic exposure control, pitch 1.375, tube rotation time of 0.7 s, matrix of 512 × 512 mm, and slice thickness of 1.25 mm. Native images were further reconstructed with a slice thickness of 0.625 mm with the same increment in lung (window level of −600 Hounsfield units (HU) and window width of 1500 HU) and mediastinum windows (window level of 40 HU and window width of 350 HU). 

Radiological lung changes were graded according to the total severity score (TSS) by Kunwei Li and CT chest score by Kunhua Li—two semi-quantitative scales widely used for COVID-19 research [6,14,15]. Each of the five pulmonary lobes was visually scored from 0 to 4 in TSS or more in detail from 0 to 5 in chest CT score. TSS involved (0) no pulmonary involvement, (1) less than 25% involvement, (2) 26–50% involvement, (3) 51–75% involvement, and (4) 76–100% involvement. The scores of all lobes were then added together to provide a total severity score ranging from 0 (no involvement) to 20 (maximum involvement) [14]. Chest CT score by on the other hand is more detailed and involved: (0) no pulmonary involvement, (1) less than 5% involvement, (2) 5–25% involvement, (3) 26–50% involvement, (4) 51–75% involvement, and (5) 76–100% involvement. The scores of all lobes were also added together to provide a chest CT score ranging from 0 (no involvement) to 25 (maximum involvement) [15]. Prevalence of persisting chest imaging abnormalities was then analyzed and correlated with clinical data. CT images were evaluated by the radiologist with 15 years of experience, blinded to patients’ clinical data.

### 2.1. Statistical Analysis

Continuous variables were presented as mean ± standard deviation (SD), whereas nominal variables were expressed as frequencies and percentages. Ordinal variables were presented by median value and the first and third quartile. To assess the relationship between ordinal variables, the non-parametric Spearman’s rank correlation was used, with a rank correlation coefficient calculated to be statistically significant for our sample size at the level of 0.317 and more. Mann–Whitney U test was used to compare CT abnormalities with sex and CT scores, as well as TSS and CT scores on onset and control CT scanning. The Fisher Exact test was used to analyze the categorical variables between groups. A two-tailed *p*-value < 0.05 was assumed statistically significant. Statistical analyses were performed with the SPSS software (IBM SPSS Statistics 26.0. USA).

### 2.2. Ethics Approval

The study was approved by the Institutional Review Ethical Board (no. KE-0254/231/2021) with written informed consent waived due to the retrospective nature of the study. All patients provided written informed consent for the chest CT in accordance with the national medical treatment regulations.

## 3. Results

In total, 39 convalescent patients (23 women and 16 men) were enrolled in the study, with the mean age of 58.7 (12.5) years (34–77; median 63). Overall, 16 patients (41%) were classified into the moderate group and 23 (59%) patients into the severe group of the disease (Table 1). The occurrence of chronic states or diseases in patients, divided into groups according to the severity of COVID-19 pneumonia, is presented in Table 2. They had their control lung CT examination performed at mean 150.6 (36.8) days and median 145 days after the onset of the disease (min–max: 107–200 days). The analysis of correlations between groups in terms of gender and severity of the disease is shown in Table 3.

### 3.1. CT Lung Abnormalities

**Figure 1:**
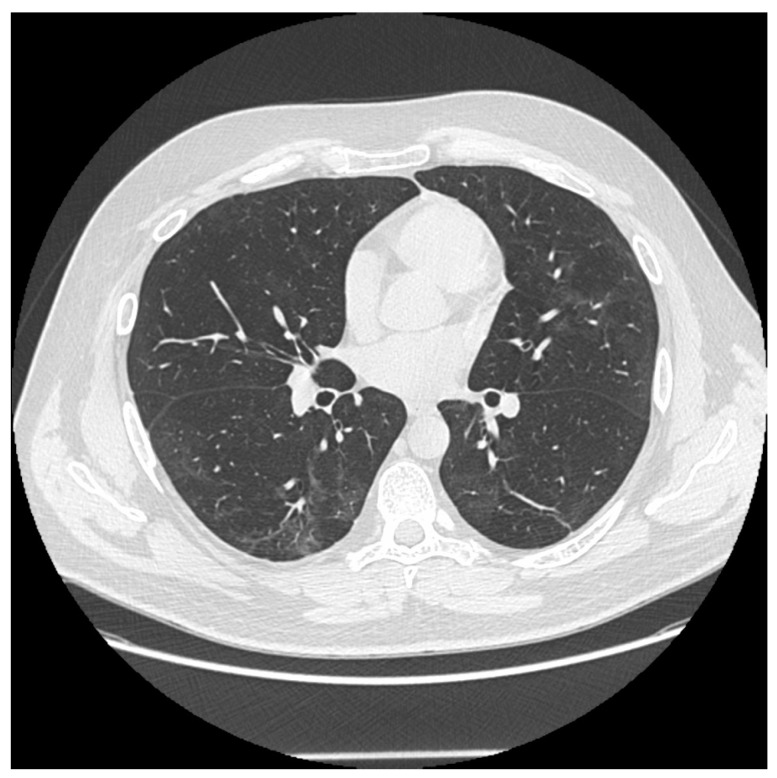
Some persistent fibrotic lines and ground glass opacities in a male patient 6 months after the onset disease.

**Figure 2:**
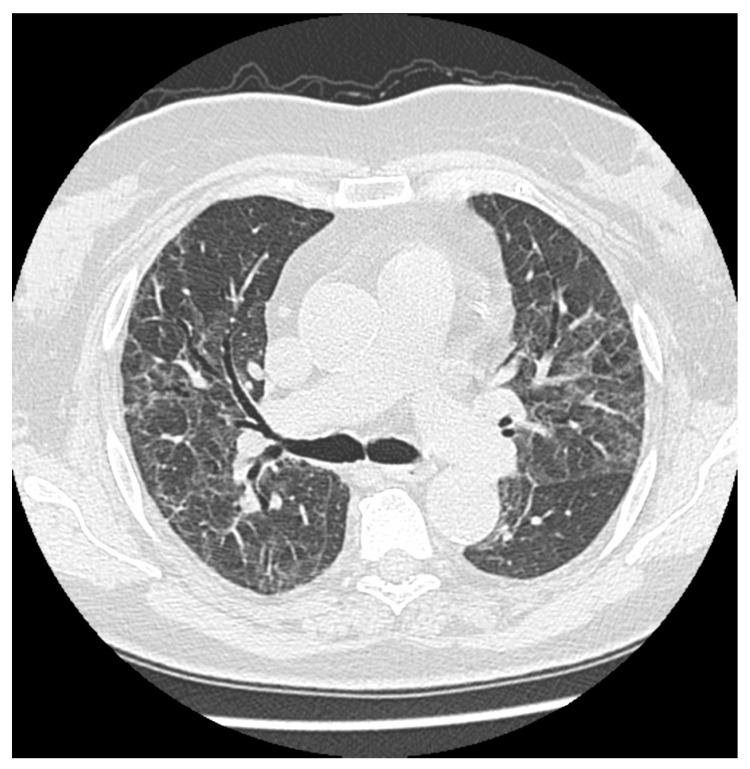
Multiple persistent ground glass opacities in a female patient 5 months after the onset of disease with singular cavitations and fibrotic lines.

On follow-up CT images, 94.9% of patients still exhibited some persistent radiological abnormalities. The most frequently observed changes at mean 5 months after COVID-19 pneumonia were ground-glass opacities (GGO) (74.4%), reticular pattern (64.1%), fibrotic changes (53.8%) (Figure 1), nodules (33.3%), bronchiectasis (15.4%), vascular enlargement (10.3%), and cavitation or air bubble signs (5.1%) (Figure 2). Table 4 shows the complete distribution of lung abnormalities in both severe and moderate groups, as well as the clinical characteristics of patients. No significant differences were noted between various sex subgroups, nor between the severe and moderate progression of the illness. However, the prevalence of reticular pattern, fibrosis, bronchiectasis, and vascular enlargement was slightly higher in the severe group of patients compared to the moderate group. 

### 3.2. CT Score Classifications

Elevated CT scores and TSS were noted in most patients 4–7 months after COVID-19 (Table 2 and Table 3). TSS was calculated at a median score of 4.1 points and interquartile range 3 (min–max: 0–11). Furthermore, the results of the chest CT score were a median of 5.4 points with interquartile range of 4.5 (min–max: 0–18). Both TSS and CT scores were analyzed compared to clinical patients’ data and the course of COVID-19. We did not observe a statistical difference between age, sex, and radiological scores—TSS and CT score (*p* = 0.32 and *p* = 0.288, respectively). Age and sex were not statistically linked with the severity of the initial disease either (*p* = 0.61 and *p* = 0.75, respectively). Both TSS and chest CT score were not associated with the severity of the initial disease (*p* = 0.32 and *p* = 0.77, respectively), indicating that, 5 months after the disease, pulmonary abnormalities reduced to a similar stage in both groups of severity of the disease. However, the number of days after the onset showed negative associations with radiological scores (TSS R = −0.21 *p* = 0.19; chest CT score R = −0.17 *p* = 0.31), which were not statistically important, showed that pulmonary changes (CT scores) were regressing with time in all patients, but too slowly to see a statistical difference after 5 months in different groups of severity at onset.

## 4. Discussion

Long-term consequences of surviving COVID-19 are not well-known. Concerns have been raised about possible pulmonary complications, such as fibrosis and progressive interstitial disease affecting COVID-19 pneumonia survivors [16]. Available data from MERS and SARS pandemics suggest residual abnormalities may persist for years after recovery, even though most lung changes regress after a few months [17,18]. Clinical symptoms also persist for a long time, even in patients after non-severe COVID-19 course [19]. 

Various other studies have been investigating CT changes visible months after discharge from the hospital and many of them have shown high occurrence of residual changes several months after discharge [9,10,13,19]. The most commonly observed one appears to be ground glass opacities. While GGOs may persist for several months, they tend to lose attenuation with time and eventually disappear [20]. They may appear to cover a larger lung area in HRCT imaging, which may be a part of their natural progression [21]. This is consistent with our study in which we observed the frequency of GGOs in 74.4% of non-vaccinated convalescent patients with long COVID-19. We also showed high frequency of reticular pattern, bronchiectases, and fibrosis in our study group.

Bronchiectases and parietal bands are also quite common as residual abnormalities. They often suggest first permanent fibrotic changes, they may, however, improve with time [20,21]. Bronchiectases may develop into lung cavitation and represent serious complication after COVID-19 [22]. It is important to note that not all of fibrotic-like changes represent genuine fibrosis, and some of them might represent vascular damage or temporal lung injury. Some of them may have also occurred before SARS-CoV-2 infection [23].

While lung fibrosis and pulmonary dysfunction is a much-feared complication of ARDS, majority of survivors recover lung function within a year. Nonetheless, COVID-19 patients who survived ARDS and underwent mechanical ventilation are at greater risk of residual fibrosis. Complications in the form of lung fibrosis are one of the most dangerous and challenging aspects in terms of diagnosis and treatment. In connection with the COVID-19 pandemic, an additional etiological factor has been added to the spectrum of various pulmonary fibrosis-related conditions. Although the pandemic has now lost its momentum, fibrosis-related complications continue to be observed in daily practice. Clinical experience shows that among these patients, some may have had pulmonary fibrosis induced by the SARS-CoV-2 infection during the course of pneumonia, but another possible scenario is that patients with previously unrecognized fibrotic processes may have CT findings suggestive of fibrosis attributed to complications of the acute viral illness when they become infected. This is undoubtedly a diagnostic riddle that will never be solved for some patients. Fibrosis after COVID-19 pneumonia has been observed to progress even during the recovery period, which may be related to a ventilation injury, secondary infections and comorbidities, and genetic and immunological factors [24].

Indeed, in our study we have observed an increase in prevalence of fibrotic changes compared to other studies. This might be caused by the large percentage of severe patients in our study group, who underwent mechanical ventilation during the acute phase of the disease. We also assessed remnant changes in patients who presented with long COVID-19, having complained about at least one clinical symptom after mean 5 months post COVID-19. There was no correlation between initial severity or CT scores and fibrotic-like lesions, which may be related to individual factors. However, the severe group of patients presented with a higher prevalence of reticular pattern, fibrosis, bronchiectasis, and vascular enlargement compared to moderate patients. 

Limitations to our study include a relatively small group of patients and lack of HRCT studies from before the SARS-CoV-2 infection. Another limitation of our study could be linked with a relatively large range of days passed between the onset of the disease and the control CT examination, which could possibly influence the correlations results.

## 5. Conclusions

In conclusion, most commonly reported changes in our group of non-vaccinated patients with long COVID are GGOs and fibrotic-like changes, which may yet regress in the long term. Massive or progressive fibrosis appears to be a rare but dangerous complication. More studies, after a longer observation period, are required to assess the risk.

The findings of our study emphasize the importance of the continuous monitoring of patients, especially those who experienced severe COVID-19 infection over the next 6 months and potentially beyond, particularly, in cases where lung changes do not completely resolve.

### What Is New?

- Although there are already numerous reports on persistent lung changes after COVID-19 recovery, our analysis of changes, occurring over a mean period of 5 months after moderate and severe form of the disease in non-vaccinated patients, presents not only the percentage of individual abnormalities in the radiological examination of the lungs, but also the intensity of changes assessed using TSS and chest CT score scales.
- In the aspect of residual changes, the severity of the onset disease does not matter—we stated that 5 months after the disease, pulmonary abnormalities reduced to a similar stage in both groups of severity of the disease in terms of TSS and chest CT score assessment.
- We also highlighted a significant percentage of non-vaccinated patients in whom, after mean 5 months of recovery, GGO, reticular patterns, and fibrotic changes were still present. This has important clinical implications and might raise a discussion of a possible target for anti-fibrotic treatment in the future.

## Figures and Tables

**Table 1:** Basic study participant characteristics and their classification onto severe and moderate groups.

	Men	Women	Total	Severe	Not Severe
N	16	23	39	23	16
%	41%	59%	100%	59.0%	41.0%
% of Severe	25.6%	33.3%	59.0%		

**Table 2:** Characteristics of patients in the study group concerning the occurrence of comorbidities.

Comorbidity	Moderate N (% of Patients in the Group)	Severe N (% of Patients in the Group)
Non-alcoholic liver disease	2 (12.5%)	4 (17.4%)
Bronchiectases	2 (12.5%	6 (26.08%)
Obesity	0 (0%)	2 (8.7%)
Emphysema	0 (0%)	2 (8.7%)
History of cancer treatment	0 (0%)	2 (8.7%)
Cardiovascular disease	0 (0%)	6 (26.08%)
None	12 (75%)	1 (4.3%)

**Table 3:** Further study participant characteristics and their CT scores at the control recovery CT scan with analysis of correlations between groups in terms of gender and severity of the disease.

	Men				Women			Total			Severe				Not Severe		
	Mean	SD	(Min–Max)		Mean	SD	(Min–Max)	Mean	SD	(Min–Max)	Mean	SD	(Min–Max)		Mean	SD	(Min–Max)
Age, years	56.8	14.4	(34–77)		60.1	11.2	(36–72)	58.7	12.5	(34–77)	59.4	12.8	(35–77)		57.8	12.5	(34–72)
Control CT time, days	156.1	35.5	(112–205)		146.8	37.9	(107–205)	150.6	36.8	(107–205)	145.0	33.5	(107–200)		158.7	40.7	(110–205)
	Men				Women			Total			Severe				Not Severe		
At Onset	Median	IQR	(Min–Max)	*p*	Median	IQR	(Min–Max)	Median	IQR	(Min–Max)	Median	IQR	(Min–Max)	*p*	Median	IQR	(Min–Max)
TSS	8	1.5	(5–13)	0.298	5	8	(4–17)	8	3.9	(4–17)	8	6	(4–17)	0.032 *	5.5	3.5	(4–9)
CT Chest score	12	3.5	(6–19)	0.382	9	9.5	(4–22)	12	5	(4–22)	13	7.5	(4–22)	0.012 *	8.5	4.3	(6–14)
Mean 5 months	Median	IQR	(Min–Max)	*p*	Median	IQR	(Min–Max)	Median	IQR	(Min–Max)	Median	IQR	(Min–Max)	*p*	Median	IQR	(Min–Max)
TSS	3.9	2.3	(1–7)	1	4.3	3.5	(0–11)	4.1	3.0	(0–11)	4.7	2.8	(0–7)	0.296	3.4	3.3	(1–11)
CT Chest score	5.1	4.3	(1–10)	0.656	5.6	4.5	(0–18)	5.4	4.5	(0–18)	6.0	4.8	(0–18)	0.575	4.5	3.5	(0–10)

Footnote: CT—computer tomography; CT chest score—computer tomography chest score; TSS—total severity score, * statistically important result with *p* < 0.05.

**Table 4:** Frequency of persistent radiological lung changes observed in convalescent 4–7 month-COVID-19 patients.

CT Abnormalities Occurrence	Men	Women	Total	Severe	Moderate	*p* Severity Values Related to Symptoms
Presence of radiological symptoms	100.0%	91.3%	94.9%	100.0%	87.5%	0.351
GGO	87.5%	65.2%	74.4%	73.9%	75.0%	1.000
Consolidations	12.5%	8.7%	10.3%	0.0%	25.0%	0.027 *
Crazy paving	0.0%	0.0%	0.0%	0.0%	0.0%	n/a
Reticular pattern	50.0%	73.9%	64.1%	65.2%	62.5%	1.000
Halo sign	12.5%	0%	5.1%	0%	12.5%	0.351
Atol sign	0.0%	0.0%	0.0%	0.0%	0.0%	n/a
Fibrosis	50.0%	56.5%	53.8%	56.5%	50.0%	0.752
Honey comb	12.5%	0.0%	5.1%	8.7%	0%	0.539
Air brochogram	0.0%	0.0%	0.0%	0.0%	0.0%	n/a
Brochiectasis	25.0%	8.7%	15.4%	17.4%	12.5%	1.000
Bronchial wall thickening	0.0%	0.0%	0.0%	0.0%	0.0%	n/a
Pleural effusion	0.0%	0.0%	0.0%	0.0%	0.0%	n/a
Pleural thickening	0.0%	0.0%	0.0%	0.0%	0.0%	n/a
Vascular enlargement	0.0%	17.4%	10.3%	17.4%	0.0%	0.132
Air bubble sign/cavitation	12.5%	0.0%	5.1%	8.7%	0.0%	0.539
Nodules	37.5%	30.4%	33.3%	30.4%	37.5%	0.736
Pericardial effusion	0.0%	0.0%	0.0%	0.0%	0.0%	n/a
Limfadenopathy	0.0%	0.0%	0.0%	0.0%	0.0%	n/a

Footnote: CT—computer tomography; GGO—ground glass opacities; n/a—non-applicable; *—statistically significant difference.

## Data Availability

The datasets analyzed during the current study are available from the corresponding author on reasonable request.
